# Nasotracheal Microbiota of Nestlings of Parent White storks with Different Foraging Habits in Spain

**DOI:** 10.1007/s10393-023-01626-x

**Published:** 2023-04-15

**Authors:** Idris Nasir Abdullahi, Guillermo Juárez-Fernández, Úrsula Höfle, Teresa Cardona-Cabrera, David Mínguez, Javier Pineda-Pampliega, Carmen Lozano, Myriam Zarazaga, Carmen Torres

**Affiliations:** 1 OneHealth-UR Research Group, Area of Biochemistry and Molecular Biology, University of La Rioja, Logroño, Spain; 2 SaBio (Health and Biotechnology) Research Group, Game and Wildlife Research Institute (CSIC-UCLM-JCCM), Ciudad Real, Spain; 3 Department of Biology, Lund University, Lund, Sweden; 4 Department of Biodiversity, Ecology and Evolution, Faculty of Biology, Complutense University of Madrid, Madrid, Spain

**Keywords:** White storks, Nasotracheal microbiota, *Staphylococcus sciuri*, Bacterial ecology, *S. aureus* colonization

## Abstract

Migratory storks could be vectors of transmission of bacteria of public health concern mediated by the colonization, persistence and excretion of such bacteria. This study aims to determine genera/species diversity, prevalence, and co-colonization indices of bacteria obtained from tracheal (T) and nasal (N) samples from storks in relation to exposure to point sources through foraging. One-hundred and thirty-six samples from 87 nestlings of colonies of parent white storks with different foraging habits (natural habitat and landfills) were obtained (84 T-samples and 52 N-samples) and processed. Morphologically distinct colonies (up to 12/sample) were randomly selected and identified by MALDI-TOF-MS. About 87.2% of the total 806 isolates recovered were identified: 398 from T-samples (56.6%) and 305 from N-samples (43.4%). Among identified isolates, 17 genera and 46 species of Gram-positive and Gram-negative bacteria were detected, *Staphylococcus* (58.0%) and *Enterococcus* (20.5%) being the most prevalent genera. *S. sciuri* was the most prevalent species from T (36.7%) and N (34.4%) cavities of total isolates, followed by *E. faecalis* (11.1% each from T and N), and *S. aureus* [T (6.5%), N (13.4%)]*. *Of N-samples, *E. faecium* was significantly associated with nestlings of parent storks foraging in landfills (*p* = 0.018). *S. sciuri* (*p* = 0.0034) and *M. caseolyticus* (*p* = 0.032) from T-samples were significantly higher among nestlings of parent storks foraging in natural habitats. More than 80% of bacterial species in the T and N cavities showed 1–10% co-colonization indices with one another, but few had ≥ 40% indices. *S. sciuri* and *E. faecalis* were the most frequent species identified in the stork nestlings. Moreover, they were highly colonized by other diverse and potentially pathogenic bacteria. Thus, storks could be sentinels of point sources and vehicles of bacterial transmission across the “One Health” ecosystems.

**Supplementary Information:**

The online version contains supplementary material available at 10.1007/s10393-023-01626-x.

## Introduction

The recent focus on the ‘One Health’ framework of public health research includes wildlife, with special reference to migratory birds that could serve as carriers and vehicles of important zoonotic bacteria of great concern to human and animal health (Abdullahi et al. 2021).

From an epidemiologist’s perspective, close contact of birds with human housing through nesting and perching and cultivated land through foraging and resting offers manifold possible transmission routes for infectious agents. In addition, species such as the white stork (*Ciconia ciconia*) that are migratory and travel between Europe and Africa could potentially mediate the transcontinental transfer of potential pathogens (Wilharm et al. 2016). It has been demonstrated that white storks are susceptible to colonization by numerous bacteria and/or infections that can have considerable direct and indirect impacts on humans, other wild, aquatic, domestic animals, livestock and the environment (Ruiz-Ripa et al. 2020; Jarma et al. 2021).

*Staphylococcus* is considered a common colonizer of the skin, peritoneum and nasotracheal cavities of many wild animals (Ruiz-Ripa et al. 2020). Among the *Staphylococcus* genus, *Staphylococcus aureus* (*S. aureus*) represents the main etiological agent of human and animal infections such as superficial skin and soft tissue infections, osteomyelitis, and septicemia, among others (Taylor and Unakal 2021). Its economic importance in livestock production is mainly represented by the emergence and spread of certain antimicrobial-resistant phenotypes (such as the methicillin-resistant *S. aureus* [MRSA]) and clones that drastically reduce animal product yield, especially in dairy cattle (Iceland et al. 2014; Lozano et al. 2016).

Even though coagulase-negative staphylococci (CoNS) are usually less virulent than *S. aureus*, they have also become important nosocomial pathogens, and many species colonize the skin and mucosal linings of both humans and animals (Becker et al. 2014). Moreover, CoNS have been reported in tracheal samples of wild birds, with a high prevalence of *S. sciuri* (Ruiz-Ripa et al. 2020). Of these, multidrug- and methicillin-resistant CoNS strains were identified, highlighting the role of wild birds as carriers of antimicrobial resistance mechanisms (Ruiz-Ripa et al. 2020). Moreover, our research group previously reported a high rate (34.8%) of *S. aureus* nasotracheal carriage in white stork nestlings exposed to human residues (Gómez et al. 2016), but that study solely focused on *S. aureus.* Available microecological evidence, in recent times, has highlighted the relevance of studying the nasal and tracheal bacterial microbiota of wild animals (Peixoto et al. 2021).

Bacteria of the genus *Enterococcus,* which are considered harmless commensals in healthy animals, are often resistant to several clinically important antibiotics, and therefore serve as sentinel microorganisms for tracking trends in resistance to antimicrobials with Gram-positive activity (Nocera et al. 2021). Enterococci comprise both commensals and opportunistic pathogens that are ubiquitous in the environment. They can be isolated from soil, water, plants, wild animals, birds, and insects (Paniagua Voiro et al. 2018). Two species are of greater clinical relevance, *E. faecalis* and *E. faecium*, and they frequently acquire resistance genes for antimicrobial agents, including the so-called ‘last resort’ antimicrobial agents (such as linezolid) representing a growing public health concern (Torres et al. 2018). Aside from staphylococci and enterococci, other different bacterial genera with medical, veterinary and agricultural concerns have been detected from the nasal and tracheal cavities of wild birds, but in very few studies (Gambino et al. 2021). In this regard, it is important to highlight the previous detection of cephalosporin-resistant *Escherichia coli* (*E. coli*) in white storks from intestinal tract samples (Höfle et al. 2020). Although *E. coli* is part of the normal microbiota of the intestine, it might be translocated to other tissues or organs of an animal.

One key question for research is how certain bacterial colonization depends on ecological traits such as foraging habits and the habitat of the host (Vittecoq et al. 2016). It is therefore important to understand the bacterial diversities in nasotracheal cavities of storks in context with their foraging behaviour, habitat and movement ecology. These traits along with the persistence and quantity of excretion of such bacteria determine the potential role of this species in the spread of pathogenic bacteria.

For instance, numerous white storks have adapted to relying on landfills for foraging during migration and wintering but also foraging and resting in rice and other cereal fields (Martín-Vélez et al. 2020). Some storks have even established colonies close to landfills (Tortosa et al. 2002). During the breeding season nevertheless adult storks primarily forage close to the nest, providing an opportunity to comparatively study the impact of diet and foraging habitat on the respiratory tract microbiota of nestlings (Pineda-Pampliega et al. 2021). Thus, to investigate how the potential nasotracheal carriages of different bacterial species in storks vary across foraging habitats and between colonies, this study aims to determine the genera/species diversity, prevalence rates and co-colonization of bacterial isolates obtained from nasotracheal (NT) samples from stork nestlings from different colonies along a habitat gradient from landfill to natural habitat in Southern Spain.

## Materials and Methods

### Sample Collection, Transportation and Preservation

White stork nestlings (juvenile storks in the nest prior to fledging) were sampled in June 2021 at 45–55 days of age. Nasal and tracheal swab samples were collected from the stork nestlings from four different colonies based on the different foraging habits of their parents when raising their chicks. This study design took advantage of the fact that during the chick-raising period, parent storks are spatially bound to the nesting habitat (*i.e*. forage primarily close to the nest) and thus a clear differentiation of the habitat in which food items are foraged is possible. Also, sampling of nestlings is less invasive and logistically less challenging than the capture of adult storks and is carried out during routine ringing procedures. The storks corresponded to four different colonies with different foraging strategies (colonies 1 and 2: located and foraging in natural habitat; colonies 3 and 4: foraging in two different landfills). Nasal and tracheal samples from a total of 87 white stork nestlings were collected, which comprised 136 samples: 84 tracheal (T) and 52 nasal (N). Of these animals, 49 had both nasal and tracheal samples collected. The uneven distribution of samples was due to technical problems, as some samples could not be processed further due to contaminations. We collected at least one full set (nasal and tracheal swabs) of samples of one of the siblings in each nest.

Nestlings were extracted from the nest by gently wrapping them in a towel and lowering them to the floor by hand or in a large bag. Each bird was ringed with a metal and a PVC ring. The PVC ring is marked with a four-digit large alphanumeric code and allows identification of the individual stork from a distance using a telescope, for example during stork counts at landfills (visual recapture). Nasal swabs were obtained using sterile cotton-tipped urethral swabs that were introduced into the left nasal opening on the beak of each individual, avoiding contact with the beak surface and external border of the cavity, and softly rotated twice to touch all nasal conchae surface. For tracheal swabs, sterile cotton-tipped swabs were used and briefly inserted into the trachea avoiding contact with the oral mucosa. Swabs were transferred immediately to commercial Amies’ transport medium tubes and stored at 4°C until arrival at the laboratory where they were frozen immediately at − 80°C until analysis. Nestlings were returned to the nest immediately after sampling. Handling of each nestling took less than 20 min and was carried out following all applicable international, national, and/or institutional guidelines for the care and ethical use of animals, specifically directive 2010/63/EU and Spanish laws 9/2003 and 32/2007, and RD 178/2004 and RD 1201/2005. All procedures were approved by the ethical committee for animal experimentation of the University of Castilla–La Mancha and authorized by the regional government of Castilla–La Mancha (permit no.: VS/MLCE/avp_21_198).

### Bacterial Isolation and Identification

The nasal and tracheal swab samples were inoculated into brain heart infusion (BHI; Condalab, Madrid, Spain) broth supplemented with 6.5% NaCl and incubated for 24 h at 37°C. After overnight incubation, the broth samples were diluted and carefully dispensed onto four different bacteria culture media: blood agar (BioMerieux), mannitol salt agar (MSA, Condalab, Madrid, Spain), oxacillin screening agar base supplemented with oxacillin (ORSAB medium, OXOID Hampshire, UK), and CHROMagar™ LIN (CHROMagar™ LIN, Paris, France). Plates were incubated for 24–48 h at 37°C, for bacterial recovery. After overnight growth, up to 12 different colonies were randomly selected per sample (based on their morphology, colour and haemolysis).

The colonies were identified by matrix-assisted laser desorption/ionization time-of-flight mass spectrometry (MALDI-TOF-MS; Bruker Daltonics, Bremen, Germany) using the standard extraction protocol recommended by the manufacturer as previously described (Torres-Sangiao et al. 2021). For the calibration of the spectrometer, the protein profile of the *E. coli* strain DH5 peptide was used (Bruker Daltonics).

### Statistical Analysis

To assess the effect of the use of landfills as a food resource on the frequency of appearance of the different bacteria, we constructed 94 linear mixed models with binomial distributed dependent variables (47 for each type of sample, nasal or tracheal). Of these, 26 were discarded because all values were equal to 0 (16 nasal and 10 tracheal). In these models, natural or landfill was included as a factor, and the nest was included as a random factor to avoid pseudo-replication. In addition, to evaluate if the presence of a microorganism differs between the nasal and tracheal cavity, 47 models with binomial distributed dependent variables were constructed. In these models, nasal or tracheal was included as a fixed factor, and nest of origin of the nestlings and natural or landfill habitat were included as random factors. Finally, to check if a correlation between the appearance of the different microorganisms exists, we calculated the Jaccard Similarity Index for all bacteria by sample type (nasal or tracheal). These models were performed in R 4.1.3 (R Core Team 2022) using the R packages lme4 (1.1–28), car (3.0–12) and vegan (2.6–2) (Bates et al. 2015; Fox and Weisberg 2019; Oksanen et al. 2022). The package ggplot2 (3.3.5) was used to create the figures (Wickham 2016). Statistical significance was set at *p* < 0.05 for all analyses.

## Results

### Frequency of Bacteria Species and Genera Recovered from the Nasal and Tracheal Samples

A total of 806 isolates were recovered (up to 12/sample), and 703 of them (87.2%) were identified by MALDI-TOF–MS: 398 from T-samples (56.6%) and 305 from N-samples (43.4%) (Table 1). A total of 17 genera and 46 species were detected. Of all the identified bacteria, 408 isolates were *Staphylococcus* (*T* = 218, *N* = 190), 144 *Enterococcus* (*T* = 74, *N* = 70), 34 *Macrococcus* (*T* = 24, *N* = 10), 30 *Bacillus* (*T* = 15, *N* = 15), 19 *Corynebacterium* (*T* = 13, *N* = 6), 22 *Proteus* (*T* = 19, *N* = 3), 11 *Lactococcus* (*T* = 9, *N* = 2), 7 *Enterobacter* (*T* = 6, *N* = 1), 3 *Arthrobacter* (*T* = 3), 6 *Streptococcus* (*T* = 4, *N* = 2), 5 *Acinetobacter* (*T* = 3, *N* = 2), 4 *Escherichia coli* (*T* = 4) and *Providencia spp* (*T* = 4), 2 *Citrobacter spp* (*N* = 2), and one each *Micrococcus spp* (*T* = 1) and *Klebsiella spp* (*N* = 1) (Table 1). Of all the bacteria genera identified, there were significant associations of *Enterococcus* and *Proteus* with the sample type collected from the storks (*N* or *T* respectively) (*p* < 0.05) (Table 1).

**Table 1:** Distribution Pattern of Bacteria Genera Identified from Tracheal and Nasal Samples of Nestling Storks Analysed.

	N^o^ (%) in tracheal samples (*n* = 85)	N^o^ (%) in nasal samples (*n* = 52)	*χ*^*2*^	*p*	Total number of isolates of		Total number of isolates in tracheal and nasal samples
					Tracheal	Nasal	
**Gram-positive cocci**							
*Staphylococcus*	76 (89.4)	51 (98.1)	3.58	0.058	218	190	408
*Enterococcus*	37 (43.5)	36 (69.2)	8.65	0.003*	74	70	144
*Macrococcus*	15 (17.6)	7 (13.5)	0.42	0.517	24	10	34
*Lactococcus*	7 (8.2)	1 (1.9)	2.24	0.126	9	2	11
*Streptococcus*	3 (3.5)	2 (3.8)	0.01	0.924	4	2	6
*Micrococcus*	0 (0.0)	1 (1.9)	2.04	0.153	0	1	1
*Vagococcus*	1 (1.2)	1 (1.9)	0.13	0.723	1	1	2
**Gram-positive bacilli**							
*Bacillus*	15 (17.6)	13 (22.4)	1.07	0.300	15	15	30
*Arthrobacter*	3 (8.2)	0 (0.0)	1.88	0.170	3	0	3
*Corynebacterium*	4 (4.7)	3 (5.8)	0.08	0.784	13	6	19
**Gram negative bacteria: Enterobacterales**							
*Proteus*	16 (18.8)	2 (3.8)	6.34	0.012*	19	3	22
*Enterobacter*	4 (4.7)	1 (1.9)	0.71	0.399	6	1	7
*Escherichia*	3 (3.5)	0 (0.0)	1.88	0.171	4	0	4
*Providencia*	4 (4.7)	0 (0.0)	2.52	0.112	4	0	4
*Klebsiella*	1 (1.2)	0 (0.0)	0.62	0.433	1	0	1
*Citrobacter*	0 (0.0)	1 (1.9)	1.62	0.204	0	2	2
**Gram negative bacteria: Non-fermenting**							
*Acinetobacter*	3 (3.5)	1 (1.9)	0.29	0.588	3	2	5
Total isolates					398	305	703

The number of viable samples from each source is as follows.

a. Both tracheal and nasal = 49.

b. Total animals tested = 87.

*Significant association determined by Chi-squared test at 95% CI.

Out of the 408 staphylococci isolates, the most frequently identified species were *S. sciuri* (*n* = 251, 61.5%), *S. aureus* (*n* = 67, 16.4%), *S. chromogenes* (*n* = 20, 5.0%), *S. epidermidis* (*n* = 17, 4.1%) and *S. xylosus* (*n* = 11, 2.7%). Out of the 144 enterococci isolates, the most frequently detected were *E. faecalis* (*n* = 78, 54.2%), *E. faecium* (*n* = 47, 32.6%), then *E. cecorum* (*n* = 8, 5.6%) and *E. casseliflavus* (*n* = 5, 3.5%) (Table 2).

**Table 2:** Number of Isolates of Each Species Recovered from the Nasal and Tracheal Samples of Nestling Storks.

Bacteria genera and species	No. (%) of isolates from tracheal samples (*n* = 85)	No. (%) of isolates from nasal samples (*n* = 52)	Total number (%) of isolates from tracheal and nasal samples	Percentage of isolates of species per genus
*Staphylococcus*				
*S. sciuri*	146 (36.7)	105 (34.4)	251 (35.7)	61.5
*S. aureus*	26 (6.5)	41 (13.4)	67 (9.5)	16.4
*S. chromogenes*	6 (1.5)	14 (4.6)	20 (2.8)	5.0
*S. epidermidis*	13 (3.3)	4 (1.3)	17 (2.4)	4.1
*S. xylosus*	2 (0.5)	9 (3.0)	11 (1.6)	2.7
*S. lentus*	7 (1.8)	3 (1.0)	10 (1.4)	2.5
*S. simulans*	1 (0.3)	7 (2.3)	8 (1.1)	1.9
*S. hominis*	7 (1.8)	0 (0.0)	7 (1.0)	1.7
*S. saprophyticus*	5 (1.3)	1 (0.3)	6 (0.9)	1.5
*S. hyicus*	1 (0.3)	4 (1.3)	5 (0.7)	0.6
*S. haemolyticus*	2 (0.5)	0 (0.0)	2 (0.3)	0.5
*S. arlettae*	0 (0.0)	2 (0.7)	2 (0.3)	0.5
*S. capitis*	1 (0.3)	0 (0.0)	1 (0.1)	0.2
*S. pasteuri*	1 (0.3)	0 (0.0)	1 (0.1)	0.2
Total	218	190	408	100.0
*Enterococcus*				
*E. faecalis*	44 (11.1)	34 (11.1)	78 (11.1)	54.2
*E. faecium*	19 (4.8)	28 (9.2)	47 (6.7)	32.6
*E. cecorum*	8 (2.0)	0 (0.0)	8 (1.1)	5.6
*E. casseliflavus*	0 (0.0)	5 (1.6)	5 (0.7)	3.5
*E. gallinarum*	1 (0.3)	1 (0.3)	2 (0.3)	1.4
*E. durans*	0 (0.0)	2 (0.7)	2 (0.3)	1.4
*E. canis*	1 (0.3)	0 (0.0)	1 (0.1)	0.7
*E. hirae*	1 (0.3)	0 (0.0)	1 (0.1)	0.7
Total	74	70	144	100.0
*Macrococcus caseolyticus*	24 (6.0)	10 (3.3)	34 (4.8)	100.0
*Lactococcus garvieae*	9 (2.3)	2 (0.7)	11 (1.6)	100.0
*Streptococcus gallolyticus*	4 (1.0)	2 (0.7)	6 (0.9)	100.0
*Micrococcus luteus*	0 (0.0)	1 (0.3)	1 (0.1)	100.0
*Vagococcus lutrae*	1 (0.3)	1 (0.3)	2 (0.3)	100.0
*Bacillus*				
*Bacillus* sp.	14 (3.5)	9 (2.9)	23 (3.3)	76.7
*B. cereus*	0 (0.0)	4 (1.3)	4 (0.6)	13.3
*B. licheniformis*	1 (0.3)	1 (0.3)	2 (0.3)	6.7
*B. subtilis*	0 (0.0)	1 (0.3)	1 (0.1)	3.3
Total	15	15	30	100.0
*Arthrobacter cretinolyticus*	3 (0.8)	0 (0.0)	3 (0.4)	100.0
*Corynebacterium*				
*Corynebacterium* sp.	12 (3.0)	3 (1.0)	15 (2.1)	78.9
*C. falsenii*	1 (0.3)	2 (0.7)	3 (0.4)	15.8
*C. auromucosum*	0 (0.0)	1 (0.3)	1 (0.1)	5.3
Total	13	6	19	100.0
*Proteus*				
*Proteus sp.*	18 (4.5)	3 (1.0)	21 (3.0)	95.5
*P. vulgaris*	1 (0.3)	0 (0.0)	1 (0.1)	4.5
Total	19	3	22	100.0
*Enterobacter*				
*E. cloacae*	6 (1.5)	0 (0.0)	6 (0.9)	85.7
*E. asburea*	0 (0.0)	1 (0.3)	1 (0.1)	14.3
Total	6	1	7	100.0
*Escherichia coli*	4 (1.0)	0 (0.0)	4 (0.6)	100.0
*Providencia*				
*P. stuartii*	3 (0.8)	0 (0.0)	3 (0.4)	75.0
*P. retgerii*	1 (0.3)	0 (0.0)	1 (0.1)	25.0
Total	4	0	4	100.0
*Klebsiella pneumoniae*	1 (0.3)	0 (0.0)	1 (0.1)	100.0
*Citrobacter*				
*C. braakii*	0 (0.0)	1 (0.3)	1 (0.1)	50.0
*C. freundii*	0 (0.0)	1 (0.3)	1 (0.1)	50.0
Total	0	2	2	100.0
*Acinetobacter*				
*A. junii*	1 (0.3)	2 (0.7)	3 (0.4)	60.0
*A. baumannii*	2 (0.5)	0 (0.0)	2 (0.3)	40.0
Total	3	2	5	100.0
Total isolates (%)	398 (56.6)	305 (43.4)	703 (100.0)	100.0

Among other genera with few species identified, *Macrococcus caseolyticus* (4.8%), *Lactococcus garvieae* (1.6%)*, Micrococcus luteus* (0.1%)*, Streptococcus gallolyticus* (0.9%)*, Arthrobacter cretinolyticus* (0.4%)*, Corynebacterium falsenii* (0.4%)*, Escherichia coli* (0.6%)*, Klebsiella pneumoniae* (0.1%) and *Acinetobacter baumannii* (0.3%) were found in low frequencies (Table 2).

### Diversity of Bacterial Species from Nasal and Tracheal Cavities of Nestlings Based on Foraging Habits of Parent Storks

Of the 52 nasal and 85 tracheal samples collected from 87 storks, about 88.1% of nestlings from parent storks foraging in natural habitats and 81.4% nestlings of parent storks foraging in landfills had at least one *Staphylococcus* sp in their tracheal samples. However, all the stork nestlings from parents foraging in natural habitats (100%) and 90.6% of those foraging in landfills had at least one *Staphylococcus* sp. in their nasal samples (Table 3).

**Table 3:** Diversity Pattern of Nasal and Tracheal Staphylococci and Enterococci of Nestlings in Relation to the Foraging Habits of Parent Storks.

Bacterial genera and species	Tracheal		Nasal	
	No. (%) of positive nestlings of parent storks foraging in natural areas	No. (%) of positive nestlings of parent storks foraging in landfills	No. (%) of positive nestlings of parent storks foraging in natural areas	No. (%) of positive nestlings of parent storks foraging in landfills
	(*n* = 42)	(*n* = 43)	(*n* = 20)	(*n* = 32)
Staphylococci	37 (88.1)	35 (81.4)	20 (100.0)	29 (90.6)
*S. sciuri*	36 (85.7)	23 (53.4)	18 (90.0)	28 (87.5)
*S. aureus*	3 (7.1)	7 (16.3)	7 (35.0)	12 (37.5)
*S. epidermidis*	0 (0.0)	8 (18.6)	1 (5.0)	4 (12.5)
*S. hominis*	0 (0.0)	7 (16.3)	0 (0.0)	0 (0.0)
*S. lentus*	2 (4.8)	4 (9.3)	1 (5.0)	3 (9.4)
*S. chromogenes*	1 (2.4)	2 (4.7)	1 (5.0)	5 (15.6)
*S. xylosus*	2 (4.8)	0 (0.0)	5 (25.0)	1 (3.1)
*S. capitis*	1 (2.4)	0 (0.0)	0 (0.0)	0 (0.0)
*S. hyicus*	0 (0.0)	1 (2.3)	0 (0.0)	0 (0.0)
*S. simulans*	0 (0.0)	7 (16.3)	2 (10.0)	2 (6.2)
*S. saprophyticus*	2 (4.8)	3 (7.0)	0 (0.0)	1 (3.1)
*S. haemolyticus*	1 (2.4)	2 (4.7)	0 (0.0)	0 (0.0)
*S. pasteuri*	1 (2.4)	0 (0.0)	0 (0.0)	0 (0.0)
*S. arlettae*	0 (0.0)	0 (0.0)	0 (0.0)	1 (3.1)
Enterococci	16 (38.1)	20 (46.5)	11 (55.0)	22 (68.8)
*E. faecalis*	10 (23.8)	10 (23.3)	8 (40.0)	11 (34.4)
*E. faecium*	5 (11.9)	8 (18.6)	1 (5.0)	14 (43.8)
*E. cecorum*	1 (2.4)	6 (13.9)	0 (0.0)	0 (0.0)
*E. canis*	1 (2.4)	0 (0.0)	0 (0.0)	0 (0.0)
*E. hirae*	1 (2.4)	0 (0.0)	0 (0.0)	0 (0.0)
*E. casseliflavus*	0 (0.0)	0 (0.0)	4 (20.0)	0 (0.0)
*E. gallinarum*	0 (0.0)	1 (2.3)	0 (0.0)	1 (3.1)
*E. durans*	0 (0.0)	0 (0.0)	1 (5.0)	0 (0.0)

NB: The number of viable tracheal and nasal samples from each group follows, respectively.

a. Foraging in natural areas = 42 T versus 20 N.

b. Foraging in landfills = 43 T versus 32 N.

On the other hand, 55.0% and 68.8% of stork nestlings from parents foraging in natural habitats and landfills, respectively, were enterococcal nasal carriers. In contrast, 38.1% and 46.5% of nestlings of parent storks foraging in natural habitats and landfills, respectively, had enterococcal tracheal carriage (Table 3).

**Figure 1:**
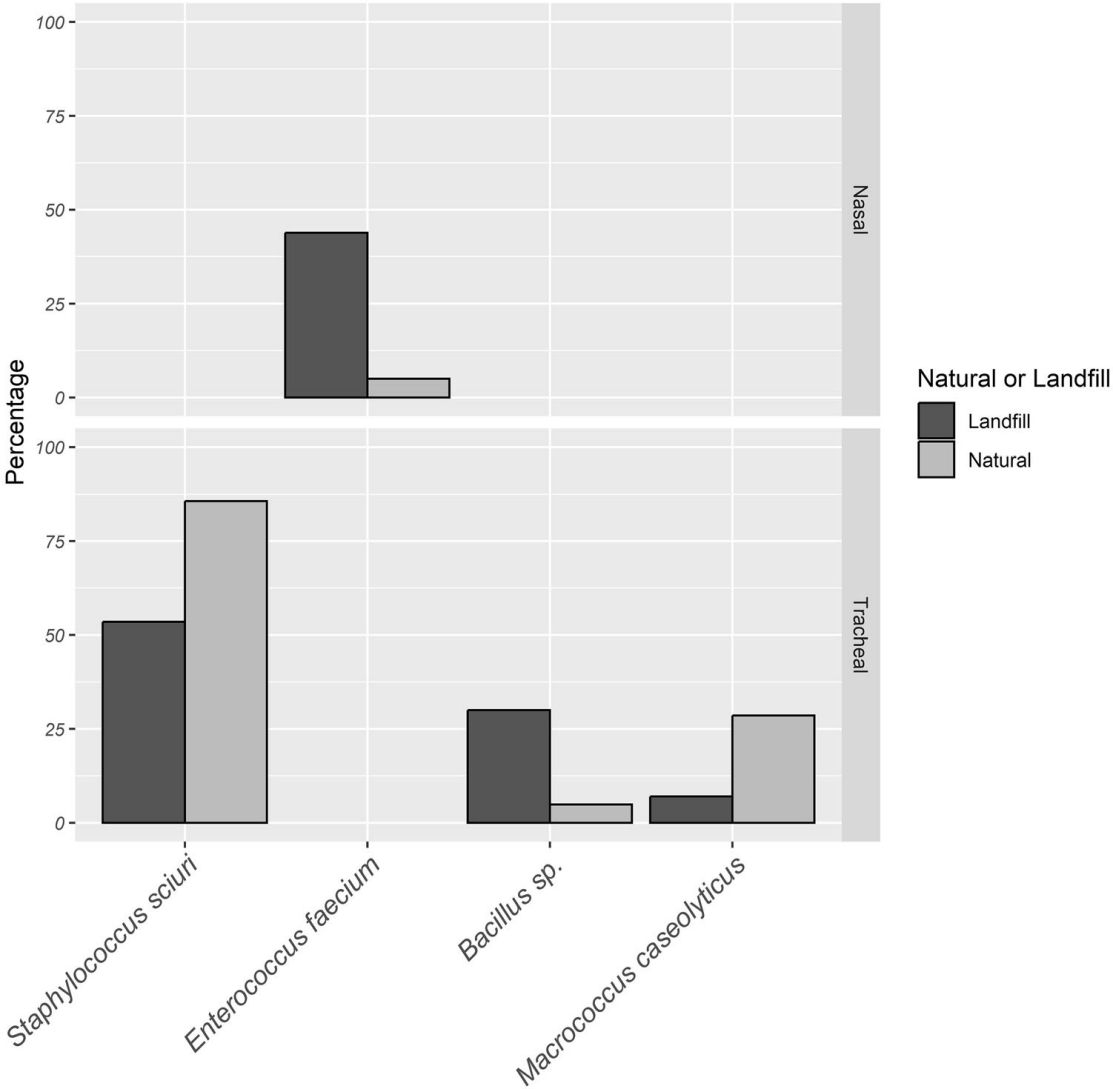
Bacterial species with significant association with foraging habitat of adults in either nasal or tracheal cavities of nestling storks.

In most cases, stork nestlings with parents foraging in landfills had a relatively higher prevalence of various species of *Staphylococcus* and *Enterococcus.* For the tracheal samples, *S. sciuri* was significantly higher among nestlings of storks foraging in natural habitats than those in landfills (*χ*^2^ = 8.568, d.f. = 1, *p* = 0.0034). In the nasal samples, a significantly higher prevalence of *E. faecium* was identified in nestlings of storks foraging in landfills than in those in the natural habitat (*χ*^*2*^ = 5.594, d.f = 1, *p* = 0.018) (Table 3, Fig. 1, Supplementary Table S1).

Regarding the other groups of bacteria in each of the samples, *M. caseolyticus *(*χ*^2^ = 4.623, d.f. = 1,* p* = 0.032) was detected significantly more frequently in the tracheal cavity of nestlings of storks foraging in natural habitat in contrast to those foraging on landfills (Fig. 1, Supplementary Table S1). In contrast, *Bacillus* sp*.* was more frequently present in samples from the tracheal cavity of nestlings of storks foraging in landfills than those in the natural habitat (*χ*^*2*^ = 8.023, d.f. = 1, *p* = 0.0046) **(**Fig. 1, Supplementary Table S1). There was no significant association between all other species identified (either from the nasal or tracheal cavity) with the foraging habits of the parent storks (Supplementary Table S1).

### Distribution Pattern of Bacterial Species Based on the Sample Types of White Stork Nestlings

**Figure 2:**
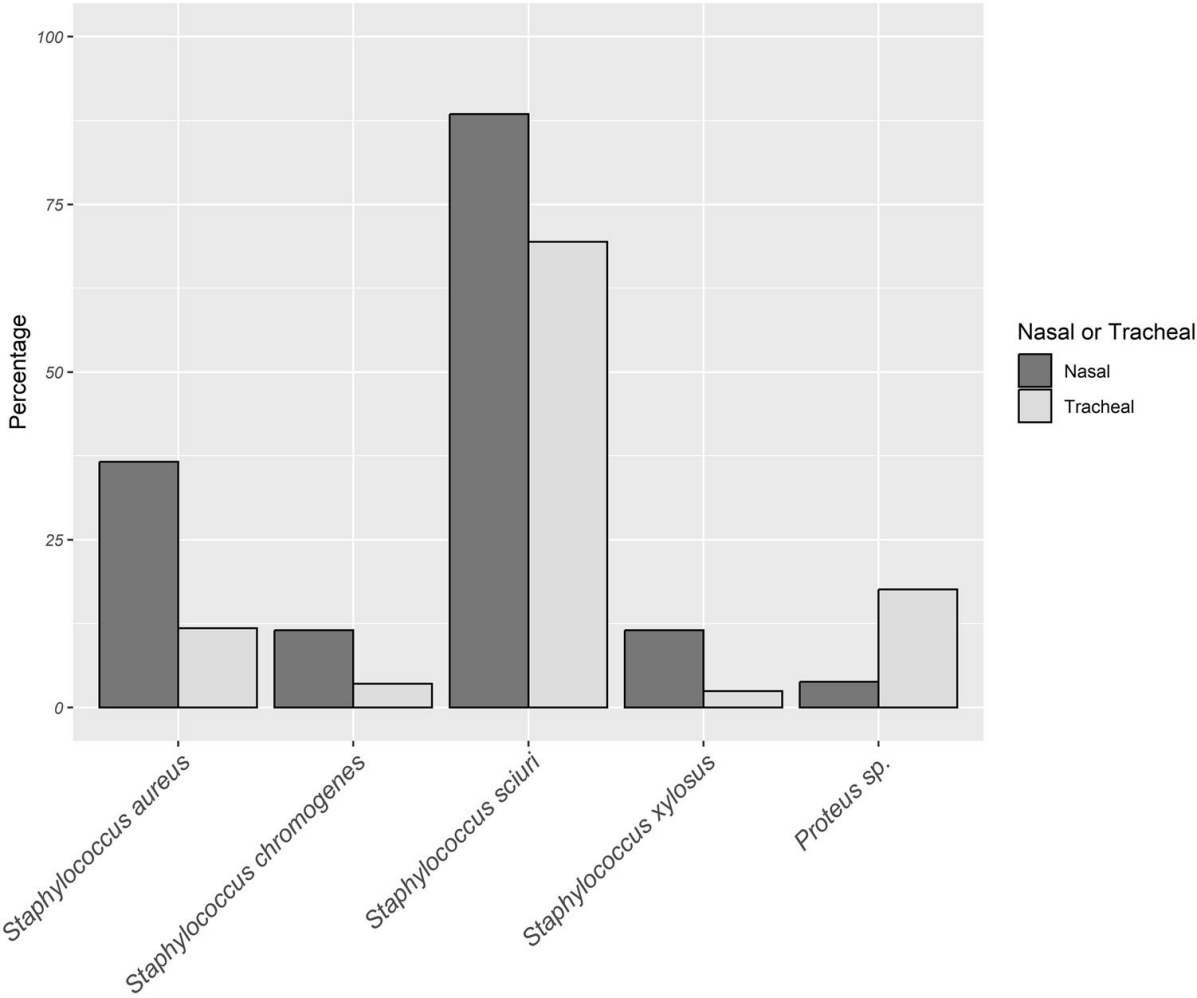
Bacterial species with significant association with sample type of nestling storks.

In most cases, the bacteria recovery rates were relatively higher from the nasal than the tracheal cavities (Supplementary Table S2). Significantly higher associations were found in *S. aureus, S. sciuri*, *S. chromogenes*, *S. xylosus* with the nasal than the tracheal cavities of the storks (*χ*^2^* test* all at d.f. = 1, *p* < 0.05, *χ* = 10.69, 6.732, 5.644 and 5.433, respectively) (Fig. 2, Supplementary Table S2). However, a significantly higher association was obtained in *Proteus* sp*.* with the tracheal than in nasal cavities of the storks (*χ*^2^ = 7.131, d.f. = 1, *p* = 0.0075) (Fig. 2, Supplementary Table S2). There was no significant association between all other species identified with the type of samples analysed (Supplementary Table S2).

### Co-Colonization of Bacteria Species in the Nasal and Tracheal Samples of White Stork Nestlings

**Figure 3:**
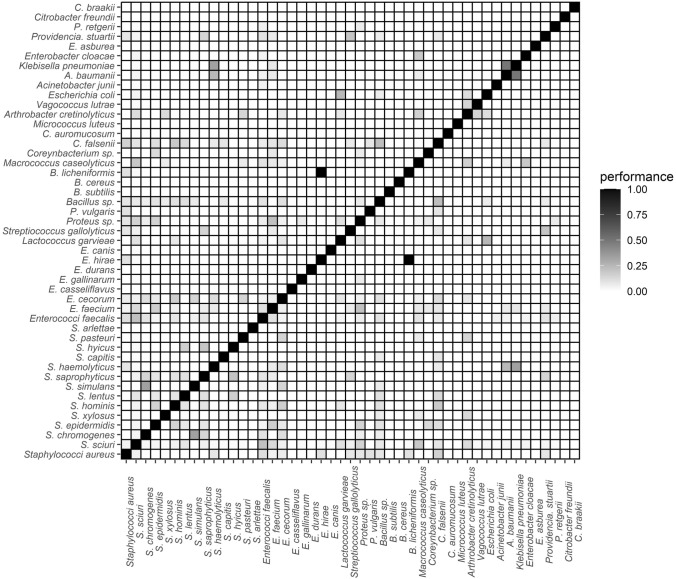
Correlation matrix of bacteria in the tracheal cavity of nestling storks. NB: The co-colonization index of two bacteria species is directly proportional to the performance level (see Supplementary Table S3).

In the tracheal cavities, the vast majority of the bacterial species had 1–10% correlation with one another (Fig. 3, Supplementary Table S3). In the remaining species, the highest correlation was between *B. lichenformis* versus *E. hirae* (100.0%), *K. pneumoniae* versus *A. baumanni* (50.0%), *S. haemolyticus* versus *K. pneumoniae* (33.3%), *A. baumanni* versus *S. haemolyticus* (25.0%) and *L. garvieae* versus *E. coli* (25.0%) (Fig. 3, Supplementary Table S3).

**Figure 4:**
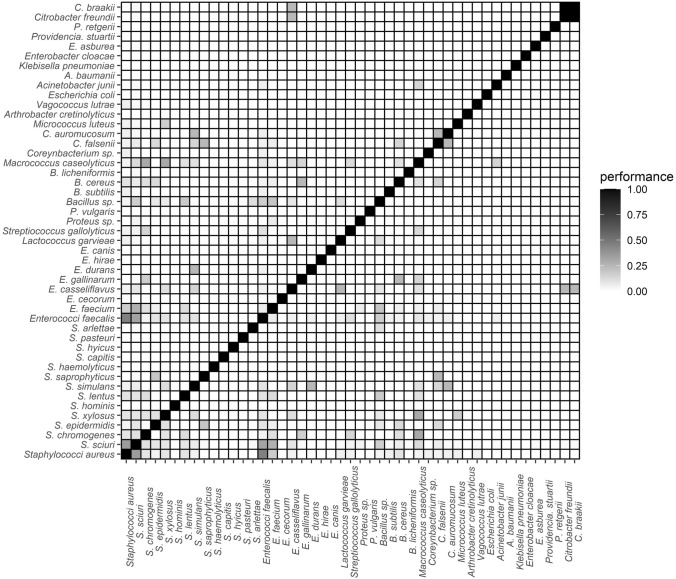
Correlation matrix of bacteria in the nasal cavity of nestling storks. NB: The co-colonization index of two bacteria species is directly proportional to the performance level (see Supplementary Table S3).

In the nasal cavities of storks, the majority of the bacterial species had 1–10% correlation between them (Fig. 4, Supplementary Table S3). In the others, the highest correlation was between *S. aureus* versus *E. faecalis* (46.2%), then *S. aureus* versus *S. sciuiri* (35.4%), *S. scuiri* versus *E. faecalis* (32.7%), and *M. caseolyticus* versus *S. chromogenes* (30.0%). Those with between 20.1 and 29.9% correlation included *S. simulans* versus *E. durans* (25.0%), *S. simulans* versus *C. auromucosum* (25.0%), *S. saprophyticus* versus *S. falsenii* (25.0%), *S. sciuri* versus *E. faecium* (27.1%), *L. garvieae* versus *E. casseliflavus* (25.0%), *E. casseliflavus* versus *C. freundii* (25.0%), and *E. casseliflavus* versus *C. braakii* (25.0%) (Fig. 4, Supplementary Table S3).

## Discussion

Migratory birds (such as storks) have been suggested to play a vital role in the spread of bacteria of public health concern across habitats and regions of the world. Key factors for a vector role are exposure to point sources of such bacteria, colonization, persistence and excretion. The former is closely related to the ecology of the species and the behaviour of individuals. In this respect, the acquisition of pathogenic bacteria through the diet (i.e. foraging) is more evident and has been reported for digestive tract samples (Wilharm et al. 2016; Höfle et al. 2020; Jarma et al. 2021). In contrast, there is a paucity of evidence for the respiratory tract to constitute a reservoir of *Staphylococcus* spp (Gómez et al. 2016). In particular, detailed bacterial diversity data on the respiratory tract and their association with the foraging habits of storks remain very scarce. Here, we report such data for nestling white storks that could also reflect the behaviour of their parents, as during the breeding season they are spatially bound to their nest, foraging primarily close to the location of the colony (Pineda-Pampliega et al. 2021).

Gram-positive cocci were the most frequently detected bacteria from the nasal and tracheal cavities of storks**,** followed by Gram-positive bacilli, while *Enterobacterales* and Gram-negative non-fermenters were relatively less frequent. Anatomically, Gram-positive cocci are often aerobic and could have a higher affinity to and colonize the upper respiratory tissues (nasal and tracheal) (Yildiz et al. 2020), and it is expected for them to be more prevalent than Gram-negative bacilli which are facultative anaerobes (such as *Enterobacterales*) and have more affinity to the intestinal lumen and tissues. This is because the gut contains low levels of oxygen due to oxygen consumption by facultative anaerobes (Franzin et al. 2021).

### Comparison of Bacteria Species by Sample Types of Nestling Storks

Even though both nasal and tracheal cavities could support the growth of most bacterial species, significant associations and higher prevalence were found in *S. aureus*, *S. sciuri*, *S. chromogenes*, and *S. xylosus* in the nasal cavities of the storks. We are unaware of any previous study that compared this phenomenon. However, a possible reason for this observation could be that the nostrils are more proximal to the external environment and more readily sustain the persistence and recovery of these bacteria (especially *S. aureus*) than the tracheal cavity. Also, it may have to do with the interactions of the staphylococci with the epithelial cells of the nose to overcome host defence mechanisms, as in the case of *S. aureus* (Sakr et al. 2018). On the other hand, *Proteus* sp*.* was significantly more frequently detected in tracheal than nasal samples. This might reflect colonization originating from the oral cavity or contamination of the sample during collection despite the care taken not to touch the oral mucosa, as *Proteus* spp. are a common inhabitant of the digestive tract.

### Comparison of Nasal and Tracheal Bacteria Carriage of Nestlings by Foraging Habits of Parent Storks

Some bacterial species were recovered in high frequencies from nestlings of parent storks foraging in landfills. The exception was *S. sciuri*, which was identified in higher frequency from the trachea of storks foraging in natural habitats. The high CoNS carriage rate detected in the nasal and tracheal samples in our storks (> 80%), is similar to the high prevalence rate previously detected in different types of wild birds in Spain (60%) (Ruiz-Ripa et al. 2020) and in Portugal (75%) (Sousa et al. 2016), but much higher than the prevalence reported in wild birds in Italy (11.4%) (Gambino et al. 2021). These differences could reflect variation in nasal and tracheal staphylococci colonization rates, the wild animal species, and could also be due to differences in methodologies used by the studies. Behavioural traits that could also influence this high prevalence could be the sharing of pastures with livestock such as cattle and small ruminants and the consumption of dung beetles by the storks, as well as the habit of storks to use cattle manure in the nest presumably to aid in the thermoregulation of newly hatched chicks (Ferreira et al. 2019; Tortosa and Villafuerte 1999). Highly diverse *Staphylococcus* spp were detected, of which *S. sciuri* and *S. aureus* accounted for over 85% of isolates of the entire genus detected. A possible explanation for the abundance of *S. sciuri* could be that this species is largely adapted to wildlife, especially wild birds, whereas *S. aureus* has a very broad host range of adaptation across various ecosystems (Guinane et al. 2010).

*Staphylococcus aureus* is a major source of opportunistic infection, especially in immunocompromised humans and a frequent etiological agent of animal infections (Haag et al. 2019). Other staphylococcal species are seldom associated with human and animal infections. It is worth mentioning that *S. scuiri* has occasionally been implicated in infections in animals (Kengkoom and Ampawong 2017; Nemeghaire et al. 2014; Zeman et al. 2017) and hospitalized humans (Cirkovic et al. 2017).

In the storks, enterococci are the second most frequent colonizers of the nasal and tracheal cavities. In this study, several non-*E. faecalis* and non-*E. faecium* species including *E. gallinarum, E. casseliflavus, E. cecorum, E. canis, E. hirae* and *E. durans* were also isolated to a small extent. It is important to mention that even though enterococci are associated with the intestinal tract of humans and animals, it was frequently detected in the NT samples of storks from our study. Specifically, *E. faecium* was significantly more frequently found in nasal samples of nestlings of adult storks foraging in landfills. The frequent nasal carriage of *E. faecium* by nestlings fed from landfills may be due to their presence in human and animal faecal-contaminated materials, for example, wastewater treatment plant sludge that is often disposed-off in landfills (EFSA 2022; Hammerum and Jensen 2002; Brendan and O’Kelly 2005).

The high prevalence of enterococci detected in our study in nasal and tracheal samples of nestling storks (43.5% and 69.2%, respectively) highlights their frequent respiratory tract carriage. There is a paucity of studies on the pathogenicity of *Enterococcus* spp from storks (wild birds). However, *E. cecorum* has previously been shown to be a facultative pathogen in birds (Jung et al. 2018). Thus, further studies on the virulence profiles of enterococci in storks could provide insights into their potential pathological effects in wild birds. Conversely, *E*. *faecalis* and *E. faecium* are clinically relevant in patients in intensive hospital care (Giacobbe et al. 2021; Kampmeier et al. 2021), while *E. cecorum* has largely been implicated in poultry infections and could affect production/yield (Souillard et al. 2022; EFSA 2022). In this regard, it is worthy to remark that about 2.4% and 13.9% of the stork nestlings fed from natural and landfill habitats in our study were *E. cecorum* tracheal carriers. This could have resulted either from *E cecorum* contamination from poultry remains or indicate that stork nestlings are natural carriers of this bacteria. Consequently, it could be important to determine if they carry virulence genes associated with pathogenic strains of this species.

The family *Enterobacteriaceae*, which was sparsely identified from the NT samples collected from the storks, could indicate that this bacteria group has more adaptations to the intestinal tract of birds as higher detections rates have previously been demonstrated by other studies on intestinal samples of storks (Wu et al. 2021; Gambino et al. 2021).

To the best of our knowledge, this study is the first to report of *M. caseolyticus, A. cretinolyticus* and *K. pneumoniae* from NT cavities of storks. *M. caseolyticus* is generally considered to be a non-pathogenic bacterium. However, a *M.* *caseolyticus* strain (*SDLY*) that caused high mortality rates has been isolated from commercial broiler chickens (Li et al. 2018). Moreover, methicillin-resistant *M. caseolyticus* strains from bovine and canine origins have been found to carry a novel *mecD* gene conferring resistance to all classes of β-lactams including anti-MRSA cephalosporins (Schwendener et al. 2017).

We are unaware of previous data on the presence of *Klebsiella* spp. in white storks, but *A. baumannii* has been reported from tracheal swabs of Polish white stork nestlings (Wilharm et al. 2017). Also, the presence of cephalosporin-resistant *Escherichia coli* was previously described from faecal samples of white storks (Höfle et al. 2020). *A. baumannii* and *K. pneumoniae* are human opportunistic pathogens and among the high-priority pathogens when they are extended-spectrum beta-lactamase and carbapenemase producers (World Health Organization 2017). Knowledge about the ecological context of pathogens is of utmost importance for elucidating their transmission pattern and developing appropriate control measures. There is a paucity of reports about the dissemination levels of certain high-priority multi-drug resistance bacteria (such as *K. pneumoniae* and *A. baumannii*) in community settings by wild animals. But, Wilharm et al (2017) reported a high detection rate of *A*. *baumannii* from storks (25% of 661) and the habitats occupied by storks.

*Klebsiella pneumoniae* and *Acinetobacter baumannii* were found in low prevalence (< 2%). Both species have been related to important infections in humans and animals (Agard et al. 2019; Kenyon 2021; Wareth and Neubauer 2021). In relation to the potential of white storks as vectors of bacteria transmission, the duration of carriage of all the bacteria species (i.e. whether transient, intermittent or permanent carriage) remains to be elucidated. Also, more detailed studies are necessary to determine if diet only or other particularities of the foraging habitat or living conditions contribute to the differences in NT bacteria of the two groups.

### Co-colonization of Bacteria Species in the Nasal and Tracheal Cavities of Storks

Animals living in highly seasonal environments adapt their diets according to changes in food availability which in turn affects the microbial communities (Xiao et al. 2019; Gong et al. 2021).

Most of the bacterial species in the nasal cavities of the stork nestlings had 1–10% correlation with one another. However, high co-colonization indices were found between a few bacteria species. By implication, the species with lower co-colonization indices suggest potential antagonism with one another through the secretion of bioactive substances (Sakr et al. 2018), highlighting the potential to harness them for biotechnological applications. For instance, some bacterial isolates are capable of secreting anti-staphylococcal molecules modulating *S. aureus* abundance (Sakr et al. 2018). Similarly, *Corynebacterium* sp. has been shown to antagonize the colonization of *S. aureus* in the nose by human cell-binding competition mechanisms (Lina et al. 2003). From our study, the correlation matrix of *S. aureus* and *Corynebacterium* spp. in the nasal and tracheal cavities was between 4.5 and 15.8%, whereas the correlation of *S. aureus* and *S. epidermidis* in the nasal and tracheal cavities was between 0.0 and 4.3% (see Supplementary Table S3). It is worthy to mention that a new *Corynebacterium* has previously been described (*Corynebacterium pelargi sp. nov.*) from the trachea of white stork nestlings (Kämpfer et al. 2015), but further study is necessary to determine if any of the unclassified *Corynebacterium* spp from our study represent this species. Some types of *S. epidermidis* seem to be capable of synthesizing the serine protease Esp that eliminates nasal *S. aureus* in healthy humans (Iwase et al. 2010), probably by degrading staphylococcal surface proteins and human receptors critical for host–pathogen interaction (Sugimoto et al. 2013). All this put together suggests that at least one bacterium could have the potency to antagonize or minimize the survival of other colonizers.

This study being a one-point sampling could not provide more data about the dynamism of nasal and tracheal carriage, colonization or persistence of the identified bacteria. Hence, this limits the categorical conclusions that can be drawn from our study. Also, it is necessary to mention that supplementation of BHI broth with 5% NaCl could suppress the growth of some halophobic (non-salt tolerant above 1%) bacteria. Hence, the bacterial community of the nasotracheal samples reported in this study might not be entirely exhaustive.

## Conclusion

This study characterized the nasal and tracheal microbiota of nestling white storks of adults with different foraging habits using MALDI-TOF-MS-based analysis. Bacterial communities of the NT cavities were highly diverse. Most of the bacterial species identified from the nasal and tracheal samples are commensals but some could become pathogenic in humans and in animals. *S. sciuri* is a very frequent bacterium in the NT cavity of storks. Also, significant variations and diversities were associated with the foraging habits of the parent storks. These results provide support to the hypothesis that storks in most anthropogenic habitats could present a higher abundance of potentially pathogenic *Enterobacteriaceae*. This is likely to be owing to the transfer of this group of bacteria from human waste. The findings will facilitate our further understanding of the relationship between biogeography, diet structure, and species diversity of the NT microbiota of white stork. Storks could be useful sentinels and should be monitored for effective control of the spread of infections of ‘One Health’ concern. Finally, although most of the bacterial species in the NT cavities of the nestling storks had 1–10% correlation levels with one another, few were ≥ 40% correlated. These results could serve as a basis for future studies in harnessing their biomedical and biotechnological applications for the control and manipulation of pathogenic bacteria in NT cavities of animals and humans.

## Supplementary Information

Below is the link to the electronic supplementary material.

Supplementary file1 (DOCX 24 kb)

Supplementary file2 (DOCX 23 kb)

Supplementary file3 (XLSX 32 kb)
