# Supplementary material for: Nasotracheal Microbiota of Nestlings of Parent White storks with Different Foraging Habits in Spain
Source: Ecohealth. 2023 Apr 15;20(1):105–21. doi: 10.1007/s10393-023-01626-x (PMC10257605; doi:10.1007/s10393-023-01626-x)
Supplement: Supplementary file 1 — Supplementary file1 (DOCX 24 kb) [file 10393_2023_1626_MOESM1_ESM.docx]

**Table S1.** Differences in the presence of each species based on foraging habitat (Natural or Landfill) and type of sample (Nasal or Tracheal)

| Species | Nasal / Tracheal | *χ^2^* (d.f.) | p-value | Presence^a^ |
| --- | --- | --- | --- | --- |
| *Staphylococcus aureus* | Nasal | 0.033 (1) | 0.855 |  |
| *S. aureus* | Tracheal | 0.0753 (1) | 0.7838 |  |
| *S. sciuri* | Nasal | 0.075 (1) | 0.784 |  |
| ***S. sciuri*** | **Tracheal** | **8.568 (1)** | **0.0034** | **Natural (86%) > Landfill (53%)** |
| *S. chromogenes* | Nasal | 0.087 (1) | 0.769 |  |
| *S. chromogenes* | Tracheal | 0.382 (1) | 0.537 |  |
| *S. epidermidis* | Nasal | 0.111 (1) | 0.739 |  |
| *S. epidermidis* | Tracheal | 0 (1) | 1 |  |
| *S. xylosus* | Nasal | 0.457 (1) | 0.499 |  |
| *S. xylosus* | Tracheal | 0.001 (1) | 0.991 |  |
| *S. hominis* | Nasal | All 0 |  |  |
| *S. hominis* | Tracheal | 0.001 (1) | 0.989 |  |
| *S. lentus* | Nasal | 0.322 (1) | 0.571 |  |
| *S. lentus* | Tracheal | 0.108 (1) | 0.743 |  |
| *S. simulans* | Nasal | 0.240 (1) | 0.625 |  |
| *S. simulans* | Tracheal | 0.001 (1) | 0.994 |  |
| *S. saprophyticus* | Nasal | 0 (1) | 0.995 |  |
| *S. saprophyticus* | Tracheal | 0.264 (1) | 0.607 |  |
| *S. haemolyticus* | Nasal | All 0 |  |  |
| *S. haemolyticus* | Tracheal | 0.001 (1) | 0.972 |  |
| *S. capitis* | Nasal | All 0 |  |  |
| *S. capitis* | Tracheal | 0.001 (1) | 0.994 |  |
| *S. hyicus* | Nasal | All 0 |  |  |
| *S. hyicus* | Tracheal | 0 (1) | 1 |  |
| *S. pasteuri* | Nasal | All 0 |  |  |
| *S. pasteuri* | Tracheal | 0.001 (1) | 0.994 |  |
| *S. arlettae* | Nasal | 0 (1) | 1 |  |
| *S. arlettae* | Tracheal | All 0 |  |  |
| *Enterococcus faecalis* | Nasal | 0.180 (1) | 0.671 |  |
| *E. faecalis* | Tracheal | 0.010 (1) | 0.919 |  |
| ***E. faecium*** | **Nasal** | **5.594 (1)** | **0.018** | **Natural (5%) < Landfill (44%)** |
| *E. faecium* | Tracheal | 0.508 (1) | 0.476 |  |
| *E. cecorum* | Nasal | All 0 |  |  |
| *E. cecorum* | Tracheal | 0.500 (1) | 0.48 |  |
| *E. casseliflavus* | Nasal | 0.001 (1) | 0.989 |  |
| *E. casseliflavus* | Tracheal | All 0 |  |  |
| *E. gallinarum* | Nasal | 0.001 (1) | 0.994 |  |
| *E. gallinarum* | Tracheal | 0.001 (1) | 0.992 |  |
| *E. durans* | Nasal | 0.001 (1) | 0.982 |  |
| *E. durans* | Tracheal | All 0 |  |  |
| *E. hirae* | Nasal | All 0 |  |  |
| *E. hirae* | Tracheal | 0.001 (1) | 0.985 |  |
| *E. canis* | Nasal | All 0 |  |  |
| *E. canis* | Tracheal | 0 (1) | 0.999 |  |
| *Lactococcus garvieae* | Nasal | 0.001 (1) | 0.982 |  |
| *L. garvieae* | Tracheal | 0.064 (1) | 0.8 |  |
| *Streptococcus gallolyticus* | Nasal | 0.004 (1) | 0.952 |  |
| *S. gallolyticus* | Tracheal | 0.001 (1) | 0.972 |  |
| *Proteus* sp*.* | Nasal | 0.115 (1) | 0.735 |  |
| *Proteus* sp*.* | Tracheal | 0.455 (1) | 0.5 |  |
| *P. vulgaris* | Nasal | All 0 |  |  |
| *P. vulgaris* | Tracheal | 0 (1) | 1 |  |
| *Bacillus* sp*.* | Nasal | 2.794 (1) | 0.095 |  |
| ***Bacillus* sp*.*** | **Tracheal** | **8.023 (1)** | **0.0046** | **Natural (5%) < Landfill (30%)** |
| *B. subtilis* | Nasal | 0 (1) | 1 |  |
| *B. subtilis* | Tracheal | All 0 |  |  |
| *B. cereus* | Nasal | 0.001 (1) | 0.989 |  |
| *B. cereus* | Tracheal | All 0 |  |  |
| *B. licheniformis* | Nasal | 0 (1) | 0.997 |  |
| *B. licheniformis* | Tracheal | 0.001 (1) | 0.985 |  |
| *Macrococcus caseolyticus* | Nasal | 0.344 (1) | 0.558 |  |
| ***M. caseolyticus*** | **Tracheal** | **4.623 (1)** | **0.032** | **Natural (29%) > Landfill (7%)** |
| *Corynebacterium* sp*.* | Nasal | 0 (1) | 1 |  |
| *Corynebacterium* sp*.* | Tracheal | 0 (1) | 1 |  |
| *C. falsenii* | Nasal | 0 (1) | 1 |  |
| *C. falsenii* | Tracheal | 1.272 (1) | 0.259 |  |
| *C. auromucosum* | Nasal | 0 (1) | 0.997 |  |
| *C. auromucosum* | Tracheal | All 0 |  |  |
| *Micrococcus luteus* | Nasal | 0.227 (1) | 0.634 |  |
| *M. luteus* | Tracheal | All 0 |  |  |
| *Arthrobacter cretinolyticus* | Nasal | All 0 |  |  |
| *A. cretinolyticus* | Tracheal | 1.091 (1) | 0.296 |  |
| *Vagococcus lutrae* | Nasal | 0.032 (1) | 0.858 |  |
| *V.lutrae* | Tracheal | 0.001 (1) | 0.987 |  |
| *Escherichia coli* | Nasal | All 0 |  |  |
| *E. coli* | Tracheal | 0.288 (1) | 0.592 |  |
| *Acinetobacter junii* | Nasal | 0 (1) | 0.999 |  |
| *A. junii* | Tracheal | 0.002 (1) | 0.967 |  |
| *A. baumannii* | Nasal | All 0 |  |  |
| *A. baumannii* | Tracheal | 0.001 (1) | 0.992 |  |
| *Klebsiella pneumoniae* | Nasal | All 0 |  |  |
| *K. pneumoniae* | Tracheal | 0.001 (1) | 0.98 |  |
| *Enterobacter cloacae* | Nasal | All 0 |  |  |
| *E. cloacae* | Tracheal | 0.872 (1) | 0.351 |  |
| *E. asburea* | Nasal | 0 (1) | 0.999 |  |
| *E. asburea* | Tracheal | All 0 |  |  |
| *Providencia stuartii* | Nasal | All 0 |  |  |
| *P. stuartii* | Tracheal | 0.001 (1) | 0.992 |  |
| *P. retgerii* | Nasal | All 0 |  |  |
| *P. retgerii* | Tracheal | 0 (1) | 1 |  |
| *Citrobacter freundii* | Nasal | 0.001 (1) | 0.988 |  |
| *C. freundii* | Tracheal | All 0 |  |  |
| *C. braakii* | Nasal | 0.001 (1) | 0.988 |  |
| *C. braakii* | Tracheal | All 0 |  |  |
