# Supplementary material for: Nasotracheal Microbiota of Nestlings of Parent White storks with Different Foraging Habits in Spain
Source: Ecohealth. 2023 Apr 15;20(1):105–21. doi: 10.1007/s10393-023-01626-x (PMC10257605; doi:10.1007/s10393-023-01626-x)
Supplement: Supplementary file 2 — Supplementary file2 (DOCX 23 kb) [file 10393_2023_1626_MOESM2_ESM.docx]

| Species | *χ^2^* (d.f.) | p-value | Presence |
| --- | --- | --- | --- |
| ***Staphylococcus aureus*** | **10.69 (1)** | **0.001** | **Nasal (37%)> Tracheal (12%)** |
| ***S. sciuri*** | **6.732 (1)** | **0.009** | **Nasal (88%)> Tracheal (69%)** |
| ***S. chromogenes*** | **5.644 (1)** | **0.019** | **Nasal (12%)> Tracheal (4%)** |
| *S. epidermidis* | 0.013 (1) | **0.909** |  |
| ***S. xylosus*** | **5.433 (1)** | **0.019** | **Nasal (12%)> Tracheal (2%)** |
| *S. hominis* | 0 (1) | 0.998 |  |
| *S. lentus* | 0.007 (1) | 0.936 |  |
| *S. simulans* | 2.842 (1) | 0.092 |  |
| *S. saprophyticus* | 1.163 (1) | 0.281 |  |
| *S. haemolyticus* | 0 (1) | 0.999 |  |
| *S. capitis* | 0 (1) | 0.999 |  |
| *S. hyicus* | 0.001 (1) | 0.97 |  |
| *S. pasteuri* | 0 (1) | 0.999 |  |
| *S. arlettae* | 0 (1) | 1 |  |
| *Enterococcus faecalis* | 2.667 (1) | 0.103 |  |
| *E. faecium* | 2.888 (1) | 0.089 |  |
| *E. cecorum* | 0 (1) | 1 |  |
| *E. casseliflavus* | 0 (1) | 1 |  |
| *E. gallinarum* | 0.004 (1) | 0.951 |  |
| *E. durans* | 0 (1) | 1 |  |
| *E. hirae* | 0 (1) | 0.999 |  |
| *E. canis* | 0 (1) | 1 |  |
| *Lactococcus garvieae* | 3.690 (1) | 0.055 |  |
| *Streptococcus gallolyticus* | 0.214 (1) | 0.644 |  |
| ***Proteus sp.*** | **7.131** | **0.008** | **Nasal (4%) < Tracheal (18%)** |
| *P. vulgaris* | 0.003 (1) | 0.956 |  |
| *Bacillus sp.* | 0.239 (1) | 0.625 |  |
| *B. subtilis* | 0 (1) | 1 |  |
| *B. cereus* | 0 (1) | 1 |  |
| *B. licheniformis* | 0.105 (1) | 0.75 |  |
| *Macrococcus caseolyticus* | 0.323 (1) | 0.57 |  |
| *Corynebacterium sp.* | 0.105 (1) | 0.746 |  |
| *C. falsenii* | 2.325 (1) | 0.127 |  |
| *C. auromucosum* | 0.001 (1) | 0.987 |  |
| *Micrococcus luteus* | 0 (1) | 1 |  |
| *Arthrobacter cretinolyticus* | 0.149 (1) | 0.7 |  |
| *Vagococcus lutrae* | 0.105 (1) | 0.75 |  |
| *Escherichia coli* | 0 (1) | 0.999 |  |
| *Acinetobacter junii* | 0.001 (1) | 0.98 |  |
| *A. baumannii* | 0.001 (1) | 0.987 |  |
| *Klebsiella pneumoniae* | 0 (1) | 0.999 |  |
| *Enterobacter cloacae* | 0 (1) | 0.998 |  |
| *E. asburea* | 0 (1) | 1 |  |
| *Providencia stuartii* | 0 (1) | 0.999 |  |
| *P. retgerii* | 0 (1) | 1 |  |
| *Citrobacter freundii* | 0 (1) | 1 |  |
| *C. braakii* | 0 (1) | 1 |  |

**Table S2.** Differences in the presence of each species based on the type of sample (Nasal or Tracheal).
